# Supplementary material for: Brain-specific lipoprotein receptors interact with astrocyte derived apolipoprotein and mediate neuron-glia lipid shuttling
Source: Nat Commun. 2021 Apr 23;12:2408. doi: 10.1038/s41467-021-22751-7 (PMC8065144; doi:10.1038/s41467-021-22751-7)
Supplement: Supplementary file 1 — Supplementary Information [file 41467_2021_22751_MOESM1_ESM.pdf]

Supplementary Figure 1

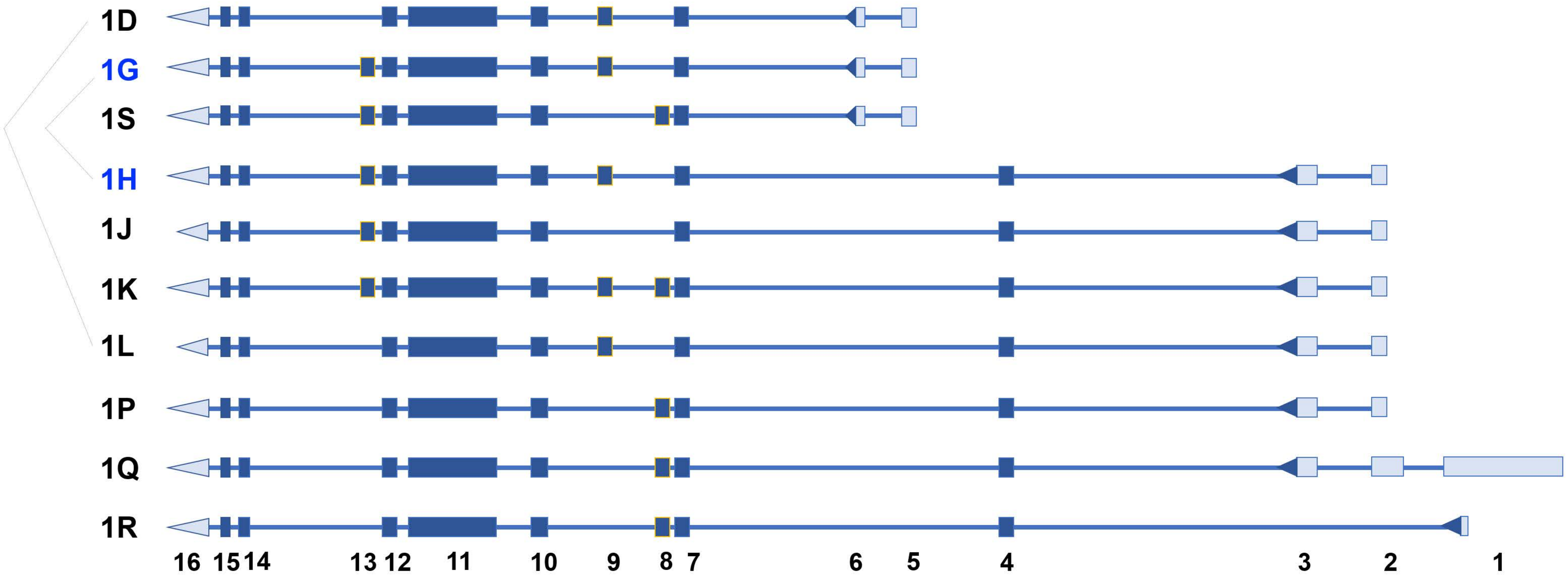

Supplementary Figure 1 A schematic diagram illustrating all *LpR1* isoforms. Untranslated regions are in light blue, coding exons are in dark blue, and flexible exons are blue with orange outlines. Two pairs of matched *LpR1-short* and *LpR1-long* isoforms are indicated by dashed lines. The *LpR1G* and *LpR1H* isoforms (highlighted in blue) are used as representatives for *LpR1-short* and *LpR1-long*.

Supplementary Figure 2

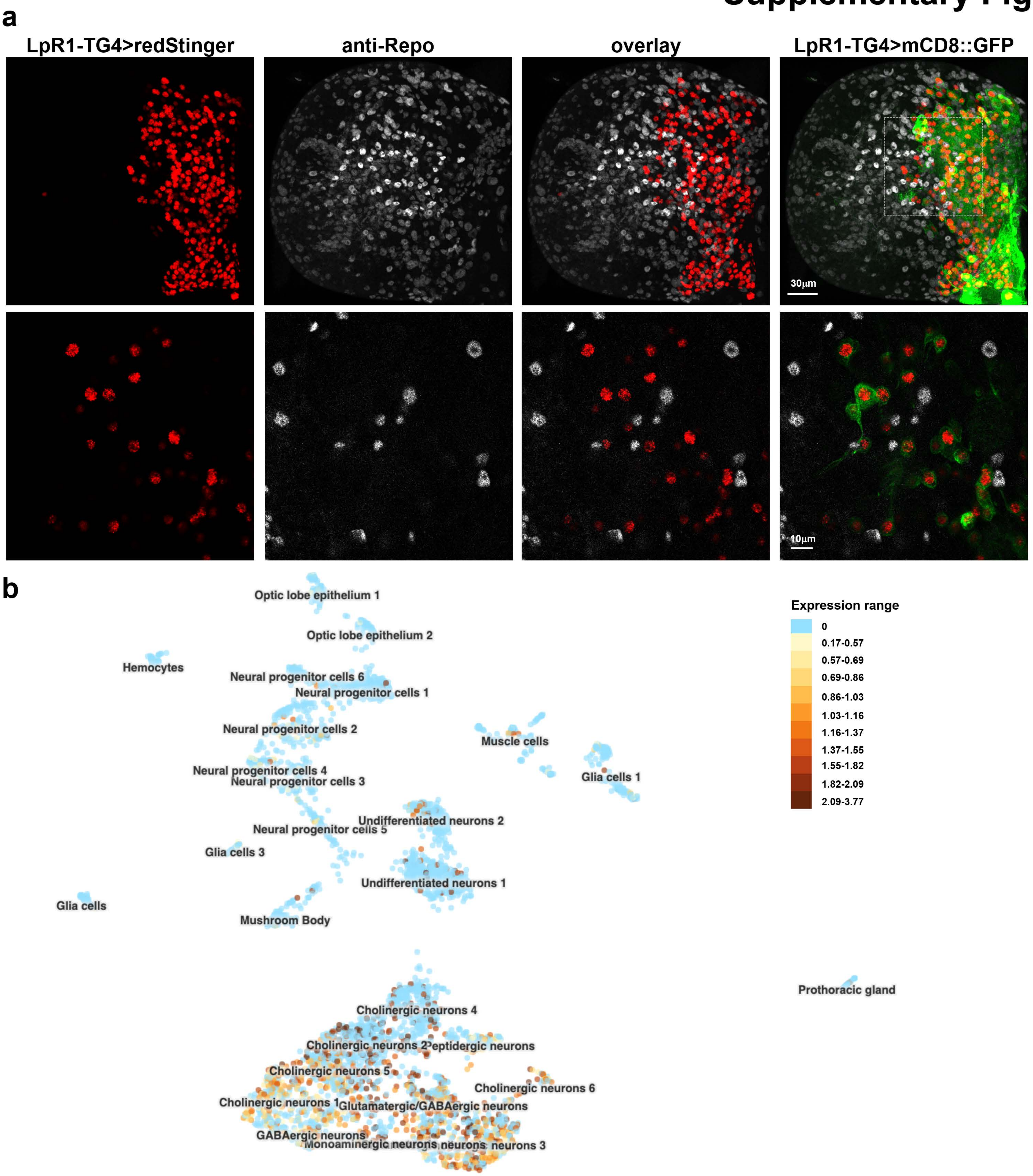

Supplementary Figure 2 LpR1 is the predominantly expressed in non-glia cells in the larval CNS. a Brain cells labeled by the LpR1 enhancer Gal4 line do not show expression of the glia marker Repo. Representative confocal images of a 3rd instar larval brain expressing redSinger and mCD8::GFP driven by LpR1-Trojan Gal4 (LpR1-TG4) and stained by anti-Repo antibody (grey) are shown (observed in at least 10 brains). Top: Maximum projection of the Z-stack of confocal images. Bottom: Single optic sections of the zoomed-in images from the area with dashed outline in the top panel. There is no grey signal found in cells labeled by LpR1-TG4 driven expression of redStinger. **b** Single cell RNA-seq data generated from the 1st instar larval brain [1] demonstrates that the expression of *LpR1* transcripts is mainly detected in the neuron clusters, including cholinergic neuron and GABAergic neuron. The color palette represents the normalized expression level range for LpR1 gene in each cell.

Supplementary Figure 3

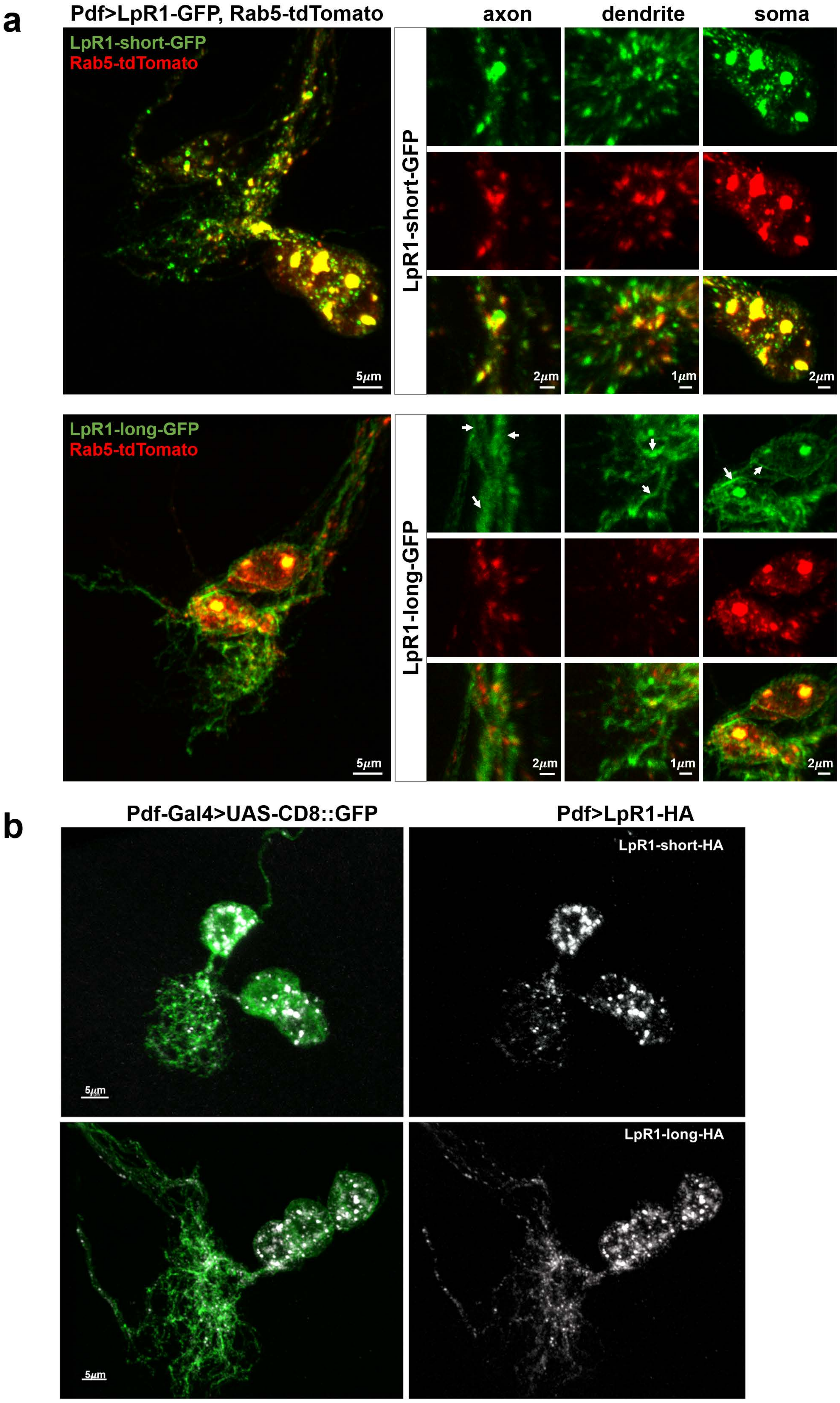

Supplementary Figure 3 LpR1-short and LpR1-long transgenes display different cellular localizations in LNvs. **a** GFP-tagged LpR1-short (LpR1-short-GFP) colocalizes with Rab5, an early endosome marker. GFP-tagged LpR1-long (LpR1-long-GFP) is also observed in vesicles but is mainly found at the cell surface and associates with membranous structures along the axonal and dendritic processes (arrows). Representative projected confocal images of LNvs expressing tdTomato tagged Rab5 (Rab5-tdTomato, red) and GFP-tagged LpR1-short or LpR1-long (LpR1-GFP, green) are shown (observed in at least 10 brains). The soma and dendritic region of LNvs (left) and zoomed-in images of axon, dendrites and soma (right) are shown. **b** The HA-tagged LpR1-short and LpR1-long are driven by LNv-specific enhancer sequence and inserted in the same genomic location by the site-specific integration (Pdf>LpR1-long-HA and Pdf>Lpr1-short-HA). The localizations of the LpR1 isoforms in the LNvs are revealed by anti-HA staining (grey) (observed in at least 10 brains), which are similar to the results obtained from the GFP-tagged LpR1 isoforms.

Supplementary Figure 4

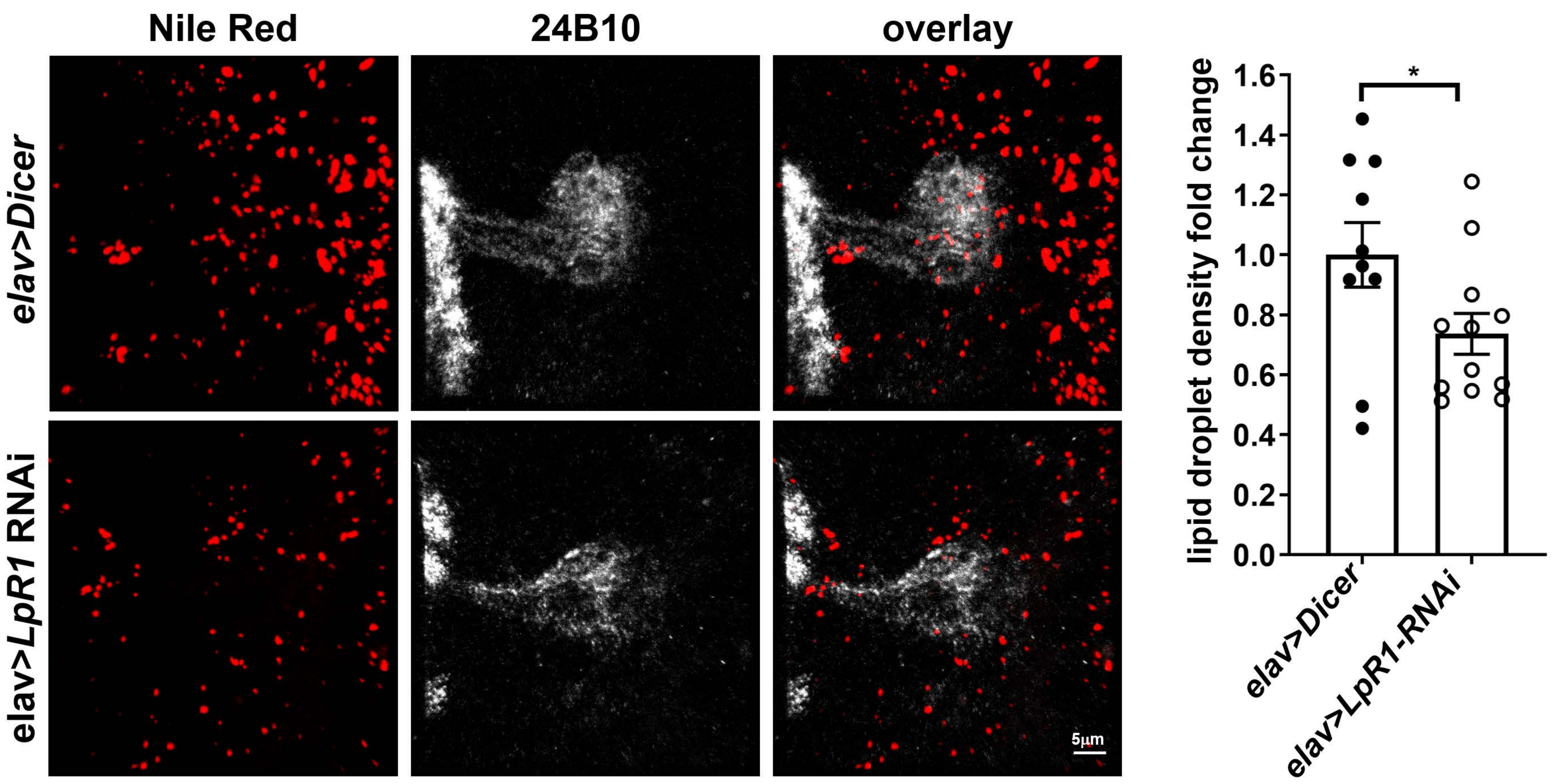

Supplementary Figure 4 *LpR1* knock-down in neurons leads to the reduction of lipid droplet density in the larval brain. Representative confocal images of lipid droplets (Nile red staining, red) and the axon terminals of photoreceptors (24B10 staining, grey) are shown (left). The lipid droplet density surrounding the LON region was quantified by 3D reconstructions (right). Data are presented as mean values +/- SEM. Statistical significance was assessed by two-tailed Student's *t*-test. $p = 0.0452$, $t = 2.136$, df = 20. n = 10 and 12 for *elav>Dicer* and *elav>LpR1-RNAi*. n represents individual larval brain sample. * $p < 0.05$.

Supplementary Figure 5

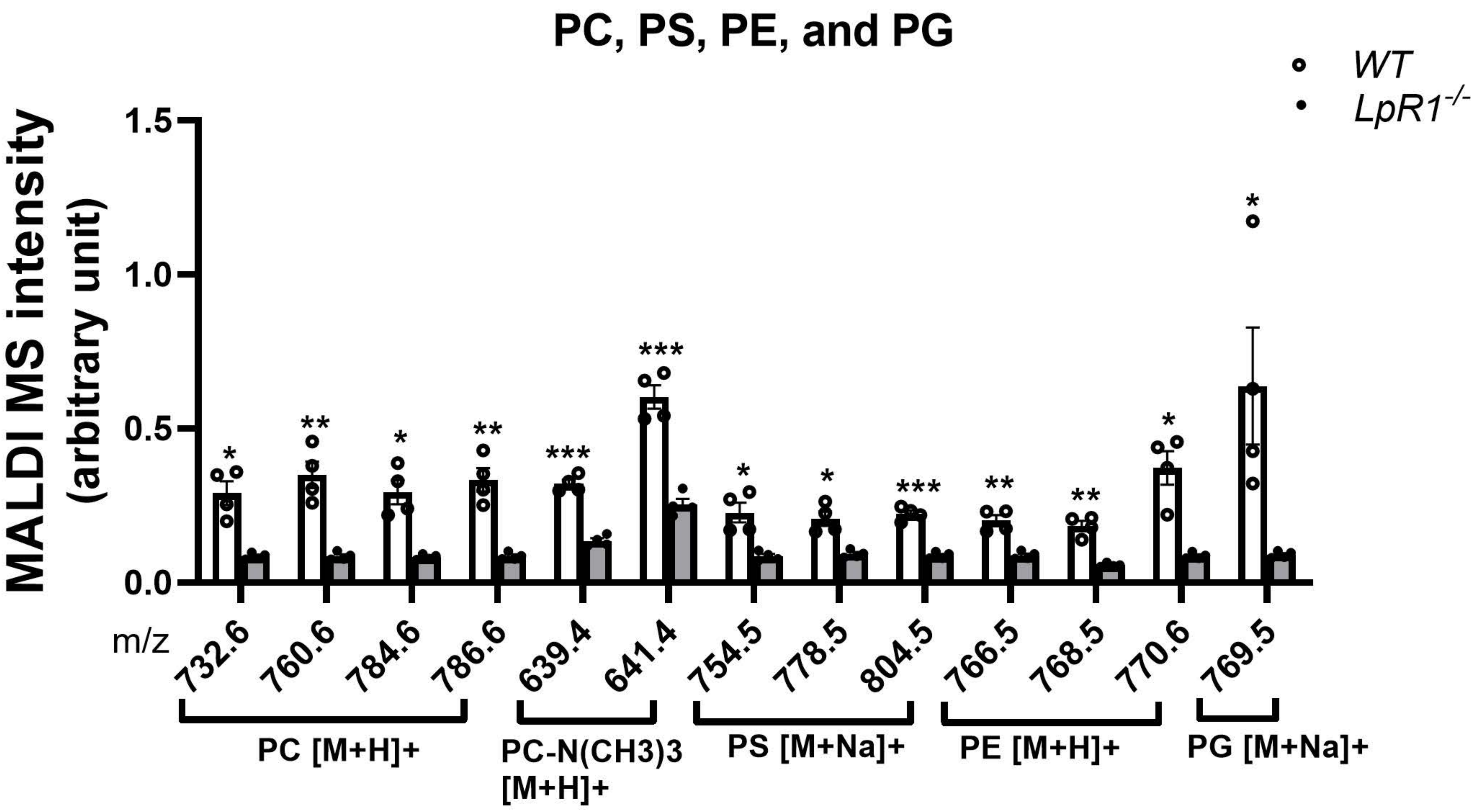

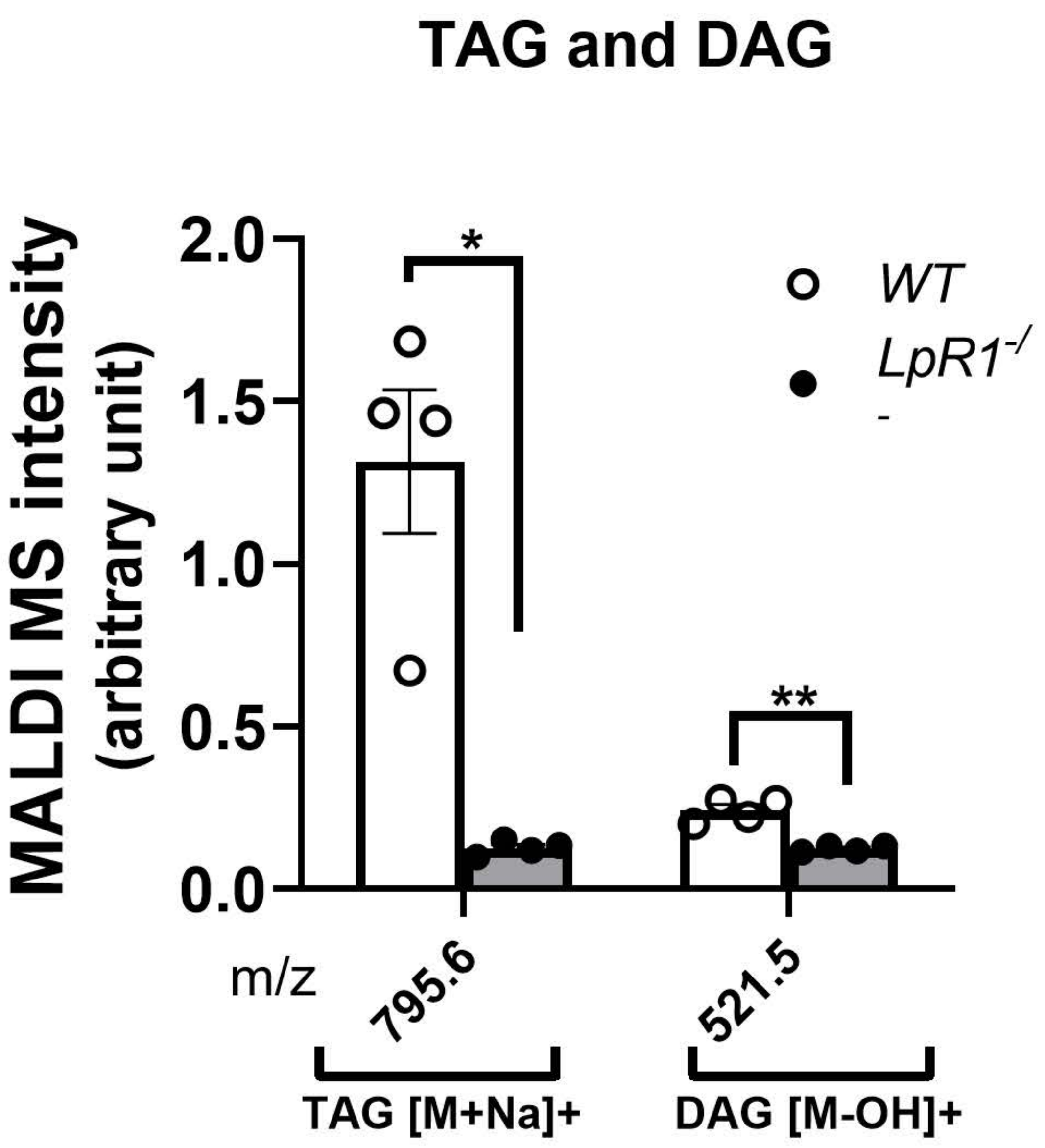

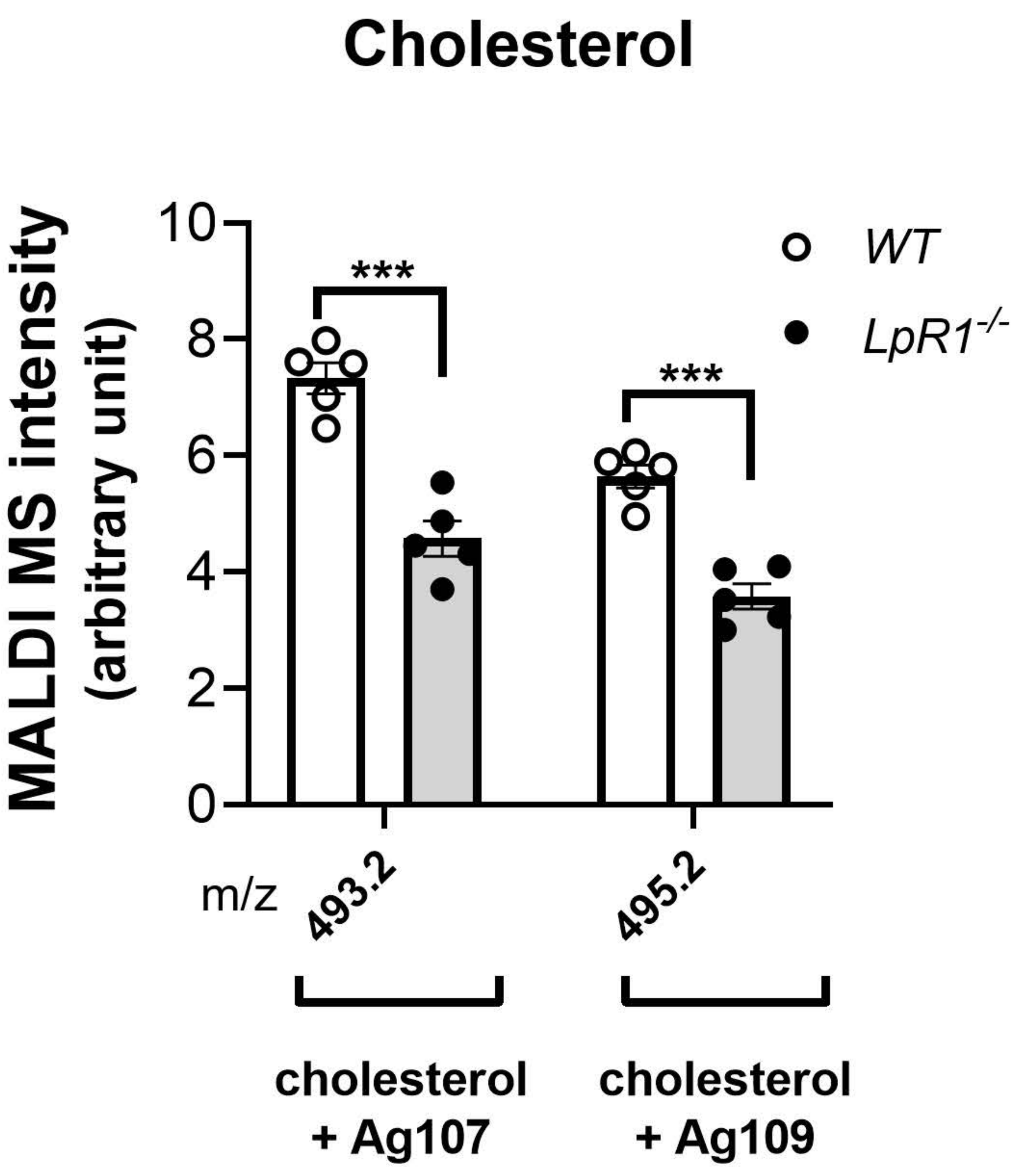

Supplementary Figure 5 MALDI Mass spectrometry analysis reveals a general reduction of brain lipid contents in *LpR1* mutants. Quantifications of MALDI imaging performed on the head sections of the adult wild type and *LpR1*$^{-/-}$ mutant flies are shown. 8 brain lipid species detected by the MALDI imaging showed significant reductions in the *LpR1*$^{-/-}$ mutant brain, as compared to the wild type control. The MALDI MS intensity value (arbitrary unit) was quantified based on scanned MALDI images of adult brain sections. Data are presented as mean values +/- SEM. Statistical significance was assessed by two-tailed Student's *t*-test. m/z-732.6: p = 0.0121; m/z-760.6: p = 0.0082; m/z-784.6: p = 0.0122; m/z-786.6: p = 0.0066; m/z-639.4: p < 0.0001; m/z-641.4: p = 0.0008; m/z-754.5: p = 0.0190; m/z-778.5: p = 0.0126; m/z-804.5: p = 0.0003; m/z- m/z-766.5: p = 0.0045; m/z-768.5: p = 0.0037; m/z-770.6: p = 0.0127; m/z-769.5: p = 0.0279; m/z-795.6: p = 0.0124; m/z-521.5: p = 0.0053. n = 4 for PC, PS, PE, PG, TAG and DAG groups. For cholesterol, m/z-493.2: p = 0.0001; m/z-495.2: p = 0.0001. n = 5 for both groups. n represents individual adult brain sample. * p < 0.05, ** p < 0.01, *** p < 0.001.

Supplementary Figure 6

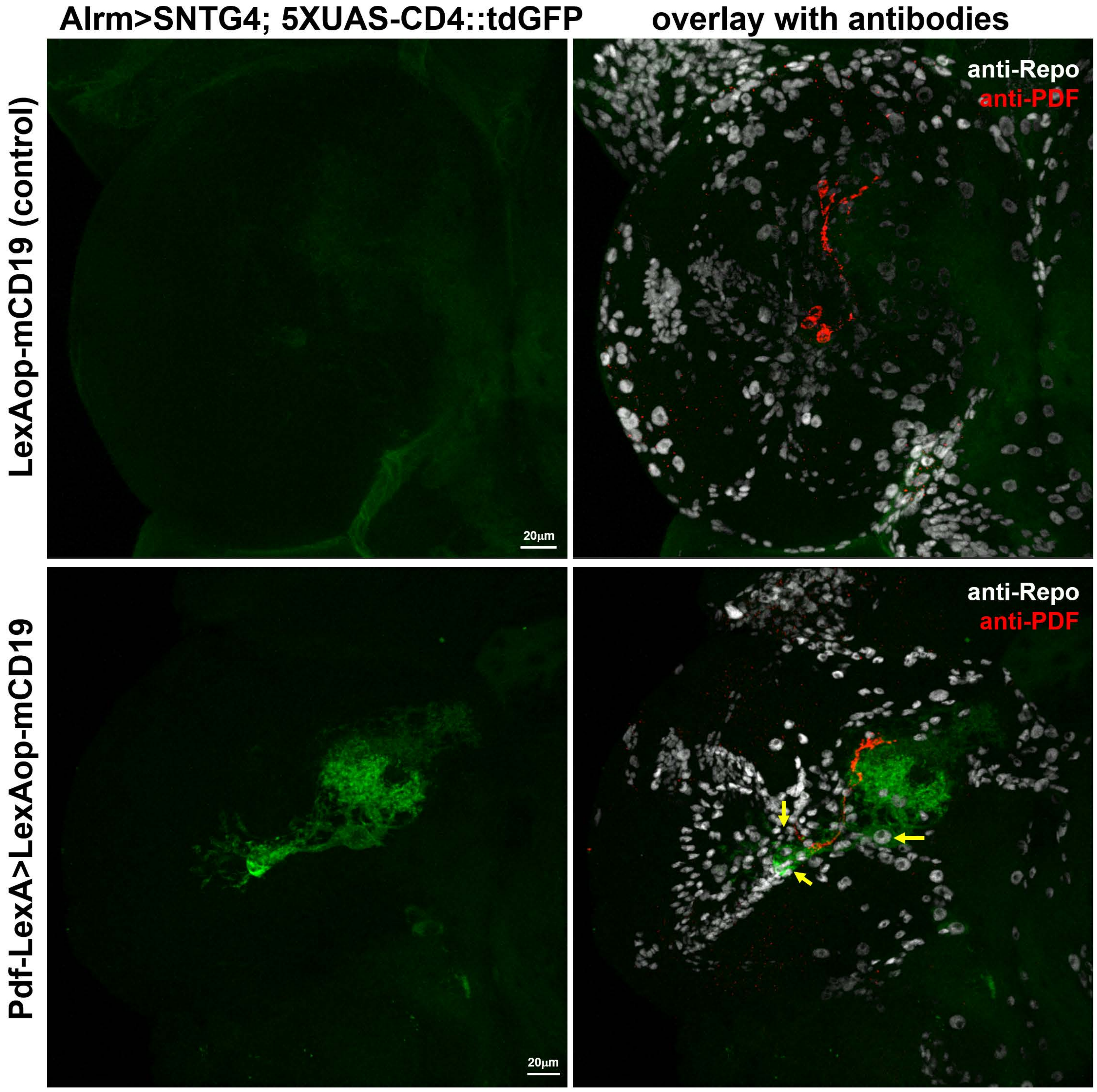

Supplementary Figure 6 Surveying astrocyte and LNv interactions using the TRAnsneuronal Control of Transcription (TRACT) technique. Interactions between LNvs and astrocytes in the 3rd instar larval brain are visualized through the induction of GFP expression in astrocytes contacting LNvs. SNTG4 receptor was expressed in astrocytes using the alrm promoter (alrm>SNTG4). When combined with the LNv-specific enhancer directed mCD19cherry expression (Pdf-LexA>Lexop-mCD19cherry), the close interaction between astrocytes and LNvs leads to the induction of GFP expression in a small set of astrocytes in the brain lobe (Green). The control group does not include the Pdf-LexA driving mCD19 expression and shows a weak background expression of GFP. Representative projected confocal images are shown (observed in at least 5 brains). The LNvs are labelled by anti-Pdf antibody (red) and nuclei of glia are labelled by anti-Repo (grey). Yellow arrows indicate the nuclei of GFP positive astrocytes.

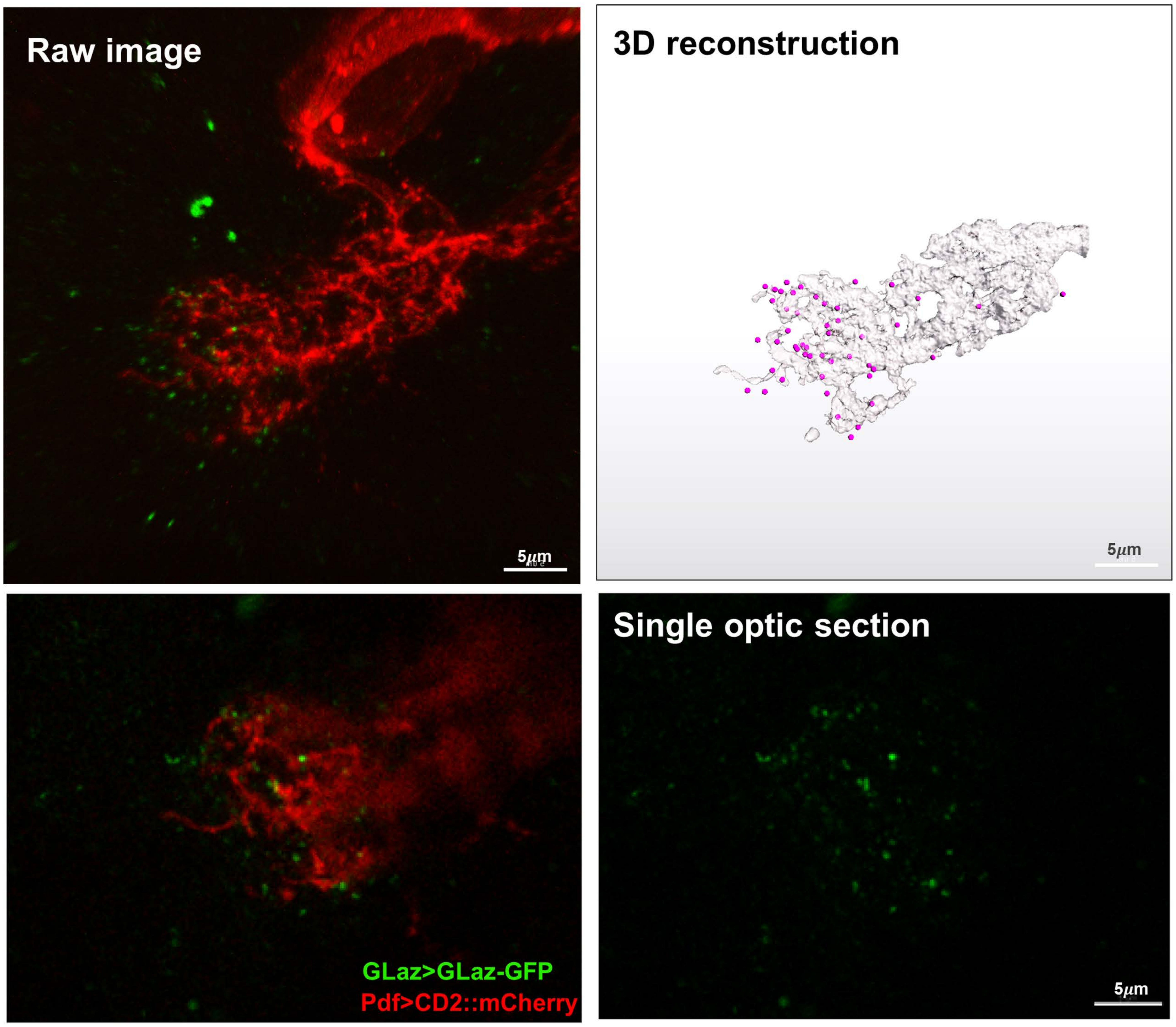

Supplementary Figure 7 The spatial relationship between GLaz-GFP and the LNv dendrite. Representative confocal images of the whole mount 3rd instar larval brain are shown (observed in at least 9 brains). LNv dendrites are labeled by Pdf-Gal4 driving CD2::mCherry. Top left: a representative projected confocal image shows that the GLaz enhancer driving GFP-tagged GLaz (GLaz>GLaz-GFP) puncta (green) are localized on the surface or in close proximity to the LNv dendrites. Top right: 3D reconstruction of the LNv dendrite (grey surface) and GLaz>GLaz-GFP puncta found within 1mm radius of the dendrite surface (magenta spots). The GFP puncta localize on the surface of the dendrites without being internalized. Bottom: a representative single optic section (0.2mm) confocal image is shown.

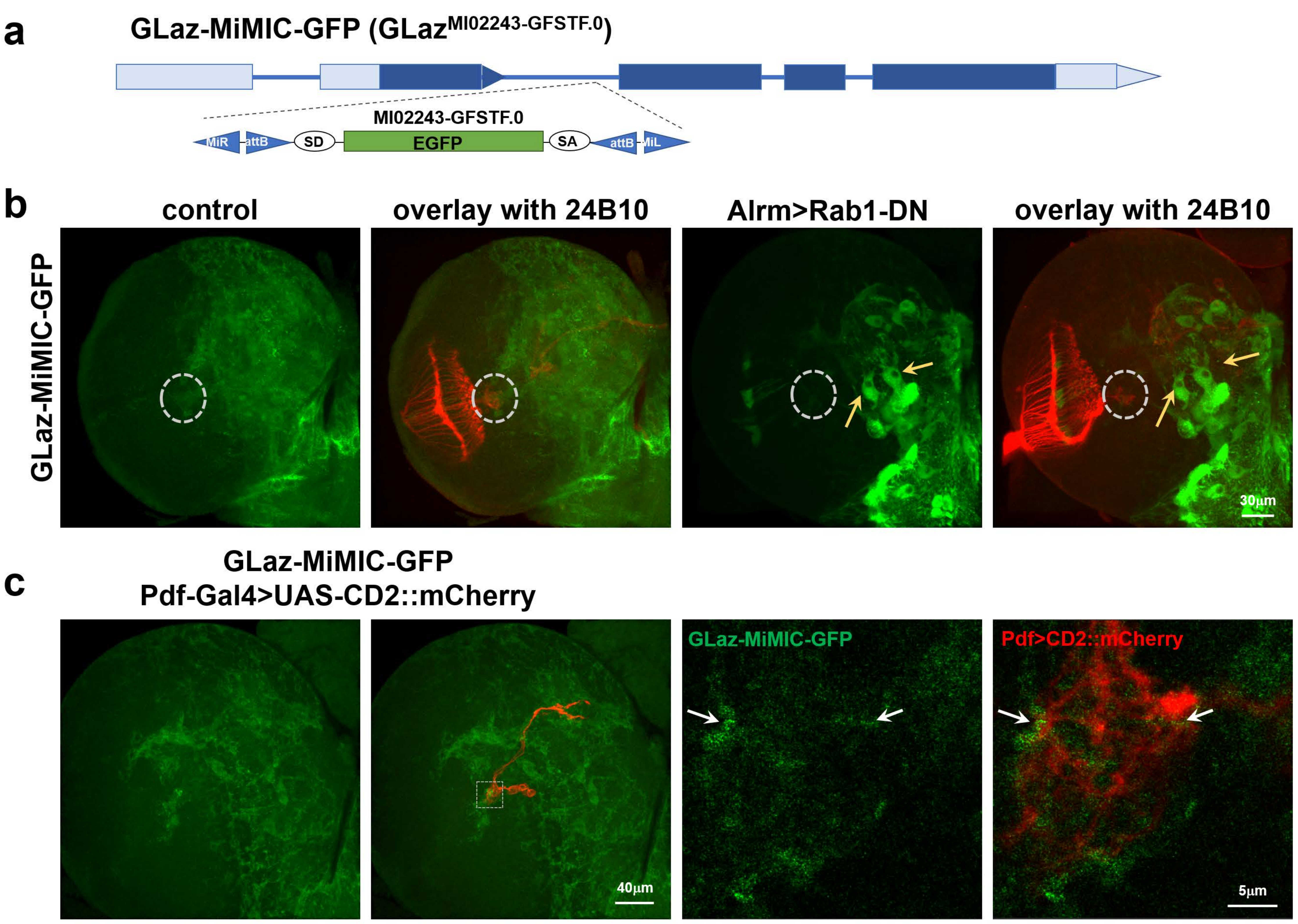

Supplementary Figure 8 GLaz is secreted from astrocytes and locates on the surface of LNv's dendrites. a A schematic diagram illustrating the protein trap line containing a GFP insertion in the GLaz protein (GLaz$^{MI02243\text{-}GFSTF.0}$). The dark blue boxes represent exons and the light blue boxes represent introns. **b** Blocking astrocytes' secretion by expressing a dominant-negative form of Rab1 (Rab1-DN) leads to the accumulation of GLaz-MiMIC-GFP (green) in somas of astrocytes (yellow arrows), in contrast to the diffused pattern observed in controls (observed in at least 10 brains). 24B10 staining (red) labels the LON (dashed circles). **c** GLaz-MiMIC-GFP signal localizes on the surface of LNv dendrites (observed in at least 10 brains). Left: Representative projected confocal images demonstrate the broad distribution of GLaz-MiMIC-GFP in the third instar larval brain. LNv dendrites are labeled by Pdf-Gal4 driven expression of CD2::mCherry. Right: Single optic sections of confocal images show that the GLaz-MiMIC-GFP signals are found close to the surface of LNv dendrites (arrows).

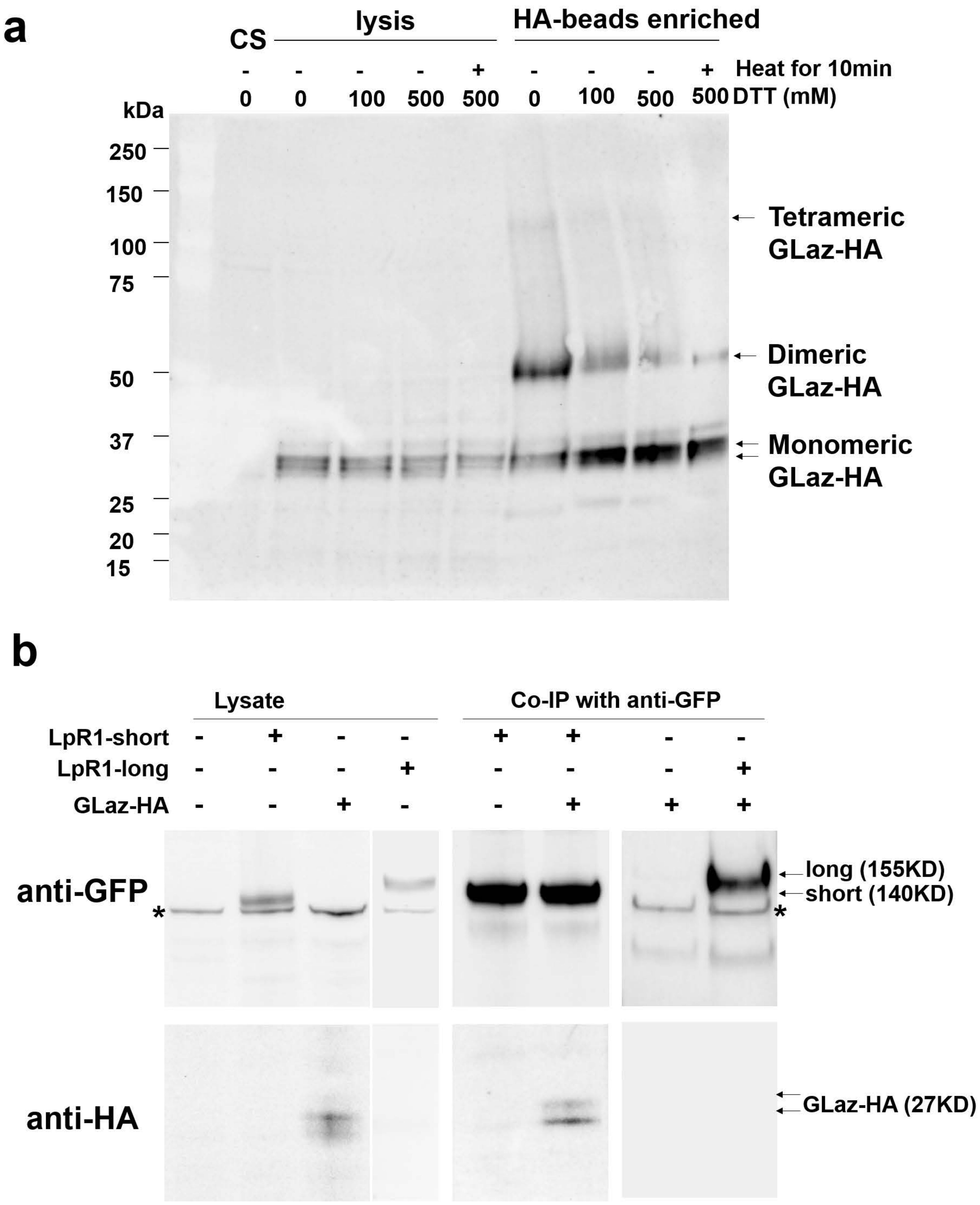

Supplementary Figure 9 GLaz protein forms dimers and tetramers and interacts directly with LpR1-short. a Dimer and tetramer forms of GLaz were observed when GLaz-HA protein was concentrated by affinity purification. The multimers were converted back to monomer by DTT and heat treatments. A representative western blot from two independent repeats using anti-HA antibody is shown. **b** Co-IP experiment using the anti-GFP beads demonstrates that LpR1-short, but not LpR1-long, binds to GLaz-HA in larval brain. Astrocyte-expressed GLaz-HA interacted with LpR1-short-GFP, but not LpR1-long-GFP, and was pulled down by the anti-GFP conjugated beads. 10% protein lysates were loaded as positive controls, in which LpR1-short-GFP (~140 KD), LpR1-long-GFP (~155 KD) and GLaz-HA (~27 KD) were detected with anti-GFP and anti-HA antibodies. * indicates a nonspecific band generated by the anti-GFP antibody. Shown is one representative result from two independent repeats.

Supplementary Figure 10

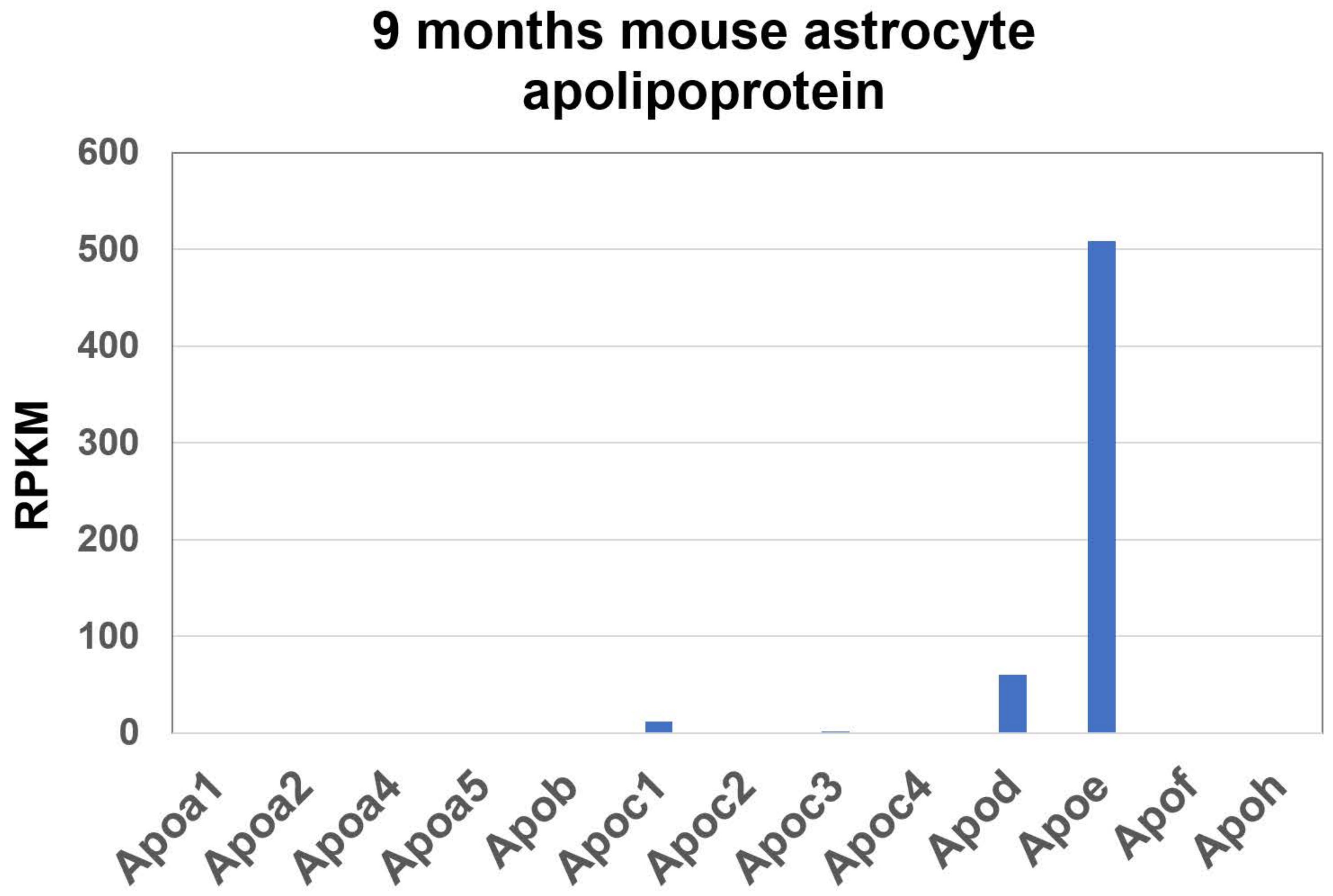

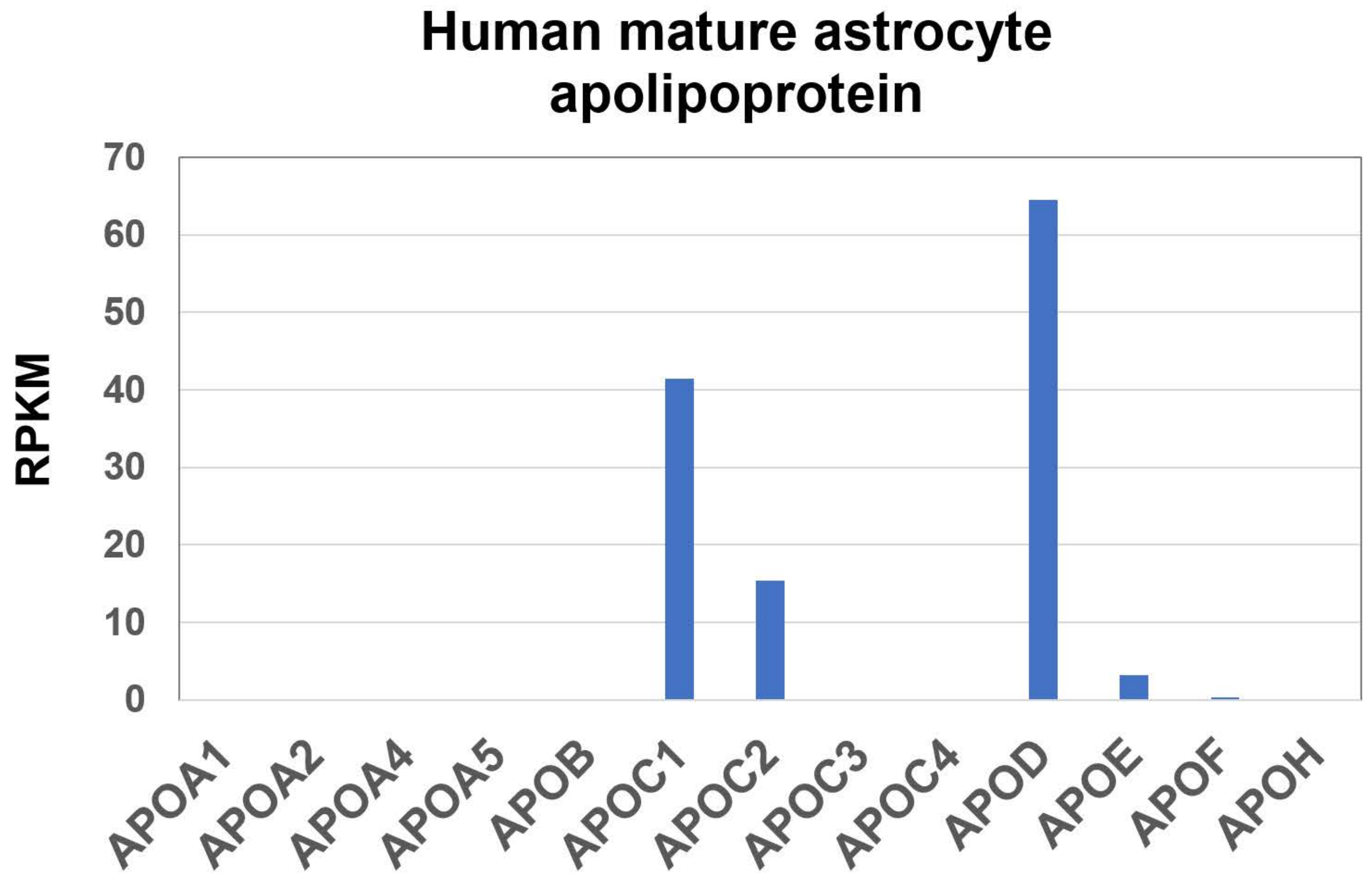

Supplementary Figure 10 APOD is highly expressed in human astrocytes. RNA-seq data reveals that APOD is the highest expressing apolipoprotein in human astrocytes. Expression levels for apolipoproteins in 9-month-old mouse astrocytes and mature human astrocytes are shown. Analyses were performed on the RNA-seq data (GEO: GSE52564) [2].

Supplementary References

1. Brunet Avalos, C., Maier, G. L., Bruggmann, R. & Sprecher, S. G. Single cell transcriptome atlas of the Drosophila larval brain. *Elife* **8**, (2019).
2. Zhang Y, *et al.* An RNA-sequencing transcriptome and splicing database of glia, neurons, and vascular cells of the cerebral cortex. *J Neurosci* **34**, 11929-11947 (2014).
