# Supplementary material for: Brain-specific lipoprotein receptors interact with astrocyte derived apolipoprotein and mediate neuron-glia lipid shuttling
Source: Nat Commun. 2021 Apr 23;12:2408. doi: 10.1038/s41467-021-22751-7 (PMC8065144; doi:10.1038/s41467-021-22751-7)
Supplement: Supplementary file 3 — Description of Additional Supplementary Files [file 41467_2021_22751_MOESM3_ESM.pdf]

Description of Additional Supplementary Files

Supplementary Data 1: The list of fly homologs for the 125 human lipid transport proteins with predicted homology scores.

Supplementary Data 2: The list of candidate Drosophila lipid transporters with their human homologs and the astrocyte enrichment levels. Astrocyte enrichment log2 fold change and p-value (by DESeq) are from Huang et al., 2015.
